# Successful atrioventricular nodal reentrant tachycardia ablation via superior vena cava approach in a patient with crisscross heart after modified Fontan with an extracardiac conduit surgery

**DOI:** 10.1016/j.hrcr.2025.06.020

**Published:** 2025-06-24

**Authors:** Masayoshi Mori, Hisaaki Aoki, Yoshihide Nakamura, Kumiyo Matsuo, Dai Asada, Yoichiro Ishii

**Affiliations:** Department of Cardiology, Osaka Women’s and Children’s Hospital, Osaka, Japan

**Keywords:** Extracardiac Fontan surgery, Catheter ablation, Crisscross heart, AVNRT, SVC approach

**Key Teaching Points**

• The superior vena cava approach can be an alternative option for ablation for the arrhythmias occurring in patients after extracardiac Fontan surgery.
• The locations of the atrioventricular node (AVN) in the crisscross heart were on the right atrial septum or 2 distinct nodes. The slow pathway could be located near the coronary sinus (CS) ostium.
• The confirmation of twin AVN and CS and fragmented potential in the vicinity of the AVN may be useful indicators for AVN reentrant tachycardia ablation.

## Introduction

Crisscross heart (CCH) is a rare congenital heart defect characterized by the abnormal crossing of 2 blood flows passing through the atrioventricular (AV) valves. It can occur in 2 forms: concordant and discordant types. In the concordant type, the inflow blood streams from the right atrium to the right ventricle (RV) and from the left atrium (LA) to the left ventricle intersect. Ventricular septal defects are frequently associated with CCH, and in the concordant type, transposition of the great arteries is also common, with the aorta originating from the RV. Patients with additional anomalies, such as hypoplastic RV, often undergo modified Fontan with an extracardiac conduit (EC) surgery. This report describes a patient with CCH who underwent a modified Fontan with an EC surgery and subsequently required ablation for AV nodal reentrant tachycardia (AVNRT). The ablation was performed by puncturing the pulmonary artery via the superior vena cava (SVC).

## Case report

**Figure 1:**
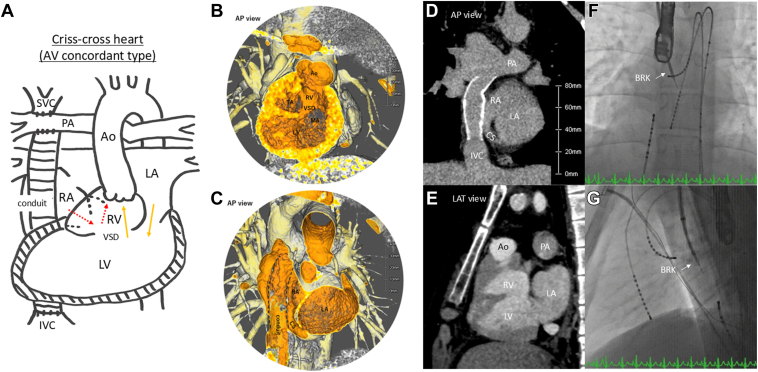
Cardiac anatomy and puncture of the PA. **A:** Simplified anatomic illustration of the AV concordance crisscross heart. The right-sided atrium drains into the RV, which gives rise to the Ao. The LA, located on the left side, drains into the LV. Blood from the LV passes through a VSD into the RV and is then ejected into the Ao. **B and C:** Computed tomography showing situs solitus and concordant crisscross heart; RV on superior and LV on inferior, TA on right superior, and MA on left inferior. A VSD is observed between the ventricles. The CS runs around the MA and drains inferior to the TA. **D and E:** Computed tomography coronal (**D**) and sagittal views (**E**) showing the positions of the IVC, conduit, PA, and LA. **F and G:** Fluoroscopy AP view (**F**) and LAT view (**G**) during puncturing of the PA to the LA using the BRK needle. Ao = aorta; AP = anteroposterior; AV = atrioventricular; CS = coronary sinus; IVC = inferior vena cava; LA = left atrium; LAT = lateral; LV = left ventricle; MA = mitral annulus; PA = pulmonary artery; RA = right atrium; RV = right ventricle; SVC = superior vena cava; TA = tricuspid annulus; VSD = ventricular septal defect.

**Figure 2:**
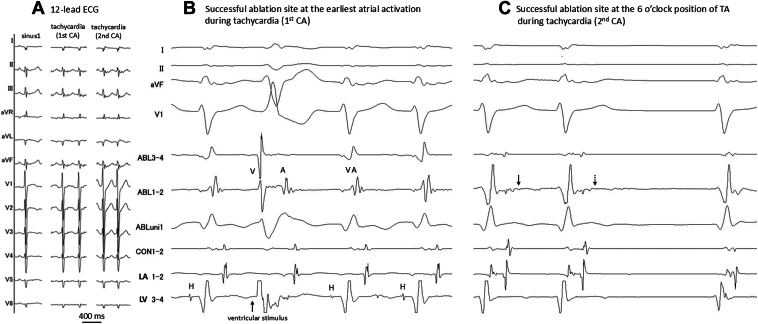
Body surface ECG and intracardiac ECG. **A:** Twelve-lead ECG of sinus rhythm and tachycardia. **B:** Intracardiac electrogram. Successful ablation site at the earliest atrial activation during tachycardia in the first session. The earliest atrial activation site was at the 1-o’clock position of the mitral annulus during tachycardia in the second session. The HA interval during tachycardia was 97 ms (this site exhibited earlier activation than the site in the first session, where the HA interval was 129 ms). Tachycardia was unaffected by the RF energy delivery at this site. **C:** Intracardiac electrogram at the successful ablation site in the second session. Fragmented atrial potentials were recorded at the 6-o’clock position of the TA during tachycardia. Tachycardia was terminated by eliminating small fragmented potentials (*arrows*) during RF application. A = atrial electrogram; ABL = ablation catheter; CA = catheter ablation; CON = conduit; ECG = electrocardiography; HA = His-atrial; LA = left atrium; LV = left ventricle; RF = radiofrequency; SVT = supraventricular tachycardia; TA = tricuspid annulus; V = ventricular electrogram.

**Figure 3:**
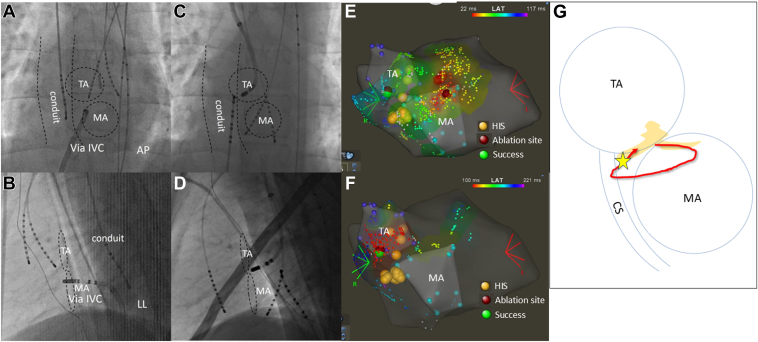
Fluoroscopy and the CARTO. **A and B:** Successful ablation sites in the first session. Ablation was performed using the IVC approach in the first session. **C and D:** Successful ablation site at the TA in the second session. **E:** Atrial activation map during the tachycardia in the second session. The earliest atrial activation occurred at the 1-o’clock position of the MA. The tachycardia was not affected by the delivery of RF energy to this site. **F:** Atrial activation map during ventricular pacing was obtained using CARTO mapping in the second session. The His electrograms were recorded at the 5-o’clock position of the TA and on the opposite side of the MA. The earliest atrial activation site was near the His recording site. **G:** Assumed circuit of AVNRT induced in the second session. The left lower extension at the 1-o’clock position of the MA was retrograded, and the right lower extension present at the 5–6-o’clock position of the TA was prograded and succeeded at the fragmented potential marked by a *star*. AP = anteroposterior; CS = coronary sinus; IVC = inferior vena cava; LAT = lateral; MA = mitral annulus; TA = tricuspid annulus.

The patient was an 18-year-old male (171 cm, 70 kg) diagnosed as having situs solitus and concordant-type CCH (Figure 1A–1E). In the present case, RV hypoplasia was observed, and biventricular repair was deemed difficult. At 1 year and 10 months of age, he underwent Glenn surgery and pulmonary artery banding, followed by modified Fontan with an EC surgery at 4 years of age using a 16-mm conduit and pulmonary artery valve closure. At the age of 12 years, he experienced palpitations (Figure 2A), for which atenolol therapy was initiated; however, the treatment was discontinued because of frequent headaches. At the age of 17 years, the first catheter ablation (CA) session was performed under general anesthesia owing to frequent palpitations. An initial attempt at conduit puncture was unsuccessful, necessitating a transcaval puncture via the inferior vena cava (IVC). This approach successfully provided access from the posterior atrial wall to the LA. His bundle potentials were identified at the 5-o’clock position of the tricuspid annulus. His potentials could not be recorded at locations other than the 5-o’clock position. During ventricular pacing, ventriculoatrial conduction demonstrated decremental properties, with the earliest activation observed at the His bundle (Figure 2B). A single atrial extrastimulus resulted in the lengthening of the atrio-His interval by more than 50 ms with a 10-ms decrease in the S2 interval (jump-up phenomenon). Tachycardia with a cycle length of 374 ms was induced and exhibited a jump-up phenomenon. The atrio-His interval was 245 ms, and the His-atrial (HA) interval was 129 ms. The earliest site of atrial activation was the His bundle, confirmed using 3-dimensional mapping. His refractory premature ventricular contraction did not reset the tachycardia. During tachycardia, ventricular overdrive pacing restored the initial V-A-V activation sequence and prolonged the ventriculoatrial time. Consequently, the supraventricular tachycardia was diagnosed as slow-slow-type AVNRT at the 5-o’clock position of the tricuspid annulus. The earliest atrial activation site during tachycardia was ablated during sinus rhythm using a 6-mm tip cryocatheter (Freezor Xtra, CryoCath, Medtronic, Montreal, Canada) (Figure 3A and 3B). Although a jump-up phenomenon was observed with atrial extrastimulation, both tachycardia and atrial echo beats could no longer be induced after ablation. However, 3 months after ablation, the patient experienced recurrent palpitations. Attempts to introduce sotalol, flecainide, amiodarone, and digoxin were unsuccessful owing to worsening fatigue and headaches.

At 19 years of age, the patient underwent a second CA session. Given the considerable difficulty encountered in achieving pulmonary atrial access via the IVC approach during the initial session, an alternative approach through the SVC was used. An 8.5F SL0 long sheath (Fast-Cath, St. Jude Medical, Inc, St. Paul, MN) was advanced via the SVC, and under fluoroscopic and transesophageal echocardiographic guidance, an attempt was made to puncture the junction between the pulmonary artery and artificial conduit using a radiofrequency (RF) needle (NRG Transseptal Needle, Baylis Medical, Montreal, Canada). However, given the approximately 13-mm distance from the pulmonary artery to the pulmonary atrium observed on computed tomography, a successful puncture could not be achieved with the RF needle. Ultimately, access was obtained using a BRK needle (St. Jude Medical, Inc) (Figure 1F and 1G). After balloon dilation of the puncture site (Sterling 4-mm- and 6-mm-diameter balloon, Boston Scientific, Tokyo, Japan), a long sheath was inserted into the atrium. The puncture procedure required 150 minutes to complete.

An electrophysiological study was performed using 3 10-pole electrode catheters: one introduced retrogradely via the aorta and positioned in the left ventricle, another advanced through the puncture site into the LA, and the third inserted via the IVC into the conduit. Ventricular extrastimulation revealed that the earliest activation site was near the His electrogram recording site at the 5-o’clock position in the tricuspid annulus. Tachycardia was induced, as described in the first session. However, the earliest atrial activation site was located at the 1-o’clock position of the mitral annulus, with an HA interval of 97 ms (this site exhibited earlier activation than the site in the first session, where the HA interval was 129 ms) (Figures 2C and 3E). His bundle potential was not recorded during sinus rhythm at this site. Tachycardia was not affected by the delivery of RF energy using a nonirrigated catheter (Navistar B-curve, Biosense Webster, Irvine, CA). However, fragmented atrial potentials were recorded at the 6-o’clock position of the tricuspid annulus during tachycardia. RF energy was then delivered in a temperature-controlled mode with a target temperature of 50°C. The energy was initially applied at 15 W and then gradually increased to 25 W. Tachycardia was successfully terminated by the elimination of small fragmented potentials during RF application (Figures 2C and 3C, 3D, and 3F). After ablation, the jump-up phenomenon disappeared during atrial extrastimulation, and tachycardia was not induced. Before the long sheath was removed, the cardiovascular surgeon was promptly contacted and instructed to remain in the catheterization room, anticipating the need for intervention in case of pericardial effusion. To manage a potential large pericardial effusion, we were prepared to use a noncompliant balloon catheter to occlude the cardiac puncture site. After the procedure, only the wire was left in the atrium during the removal of the long sheath to confirm the absence of extravascular leakage from the puncture site in the left pulmonary artery. No complications occurred. The patient remained free of recurrence over a 1.6-year follow-up period.

## Discussion

We successfully performed ablation via the SVC approach for AVNRT occurring in a patient with CCH who had previously undergone modified Fontan with an EC surgery. There have been reports of approaches from the SVC after modified Fontan with an EC surgery, with CA in 1 case^1^ and pacemaker implantation in another.^2^ In the present case, the transcaval approach via the SVC was selected for the second ablation session because of the difficulty encountered with the IVC approach in the first session. Given that the puncture site was in the pulmonary artery and atrium, an RF needle was expected to be sufficient. However, owing to approximately 20 mm of autologous tissue extending from the pulmonary artery wall to the atrium, a BRK needle was required (Supplemental Video 1). Fluoroscopy and transesophageal echocardiography were used for monitoring. Sheath insertion required balloon dilation of the puncture hole, similar to the IVC approach. A Navistar B-curve catheter was used, and its maneuverability was better than that of the IVC approach. The sheath was removed by placing a wire over the puncture hole and observing using echocardiography to confirm the absence of leakage into the epicardium before removing the wire.

In AVNRT, determining the position of the AV node is crucial to prevent AV block during treatment. Eight previous studies assessed the location of the AV node or His bundle in CCH; however, in 5 cases,^3^, ^4^, ^5^, ^6^, ^7^ the location of the slow pathway in the CCH remained unknown. The AV node was located anterior to the right atrial septum (ie, in the usual position), and in 3 cases, 2 AV nodes were identified.^8^, ^9^, ^10^ Of the twin AV node cases, 2 were situs solitus and discordant CCH^8^ and 1 was situs inversus and concordant CCH.^9^ In 2 case reports involving AVNRT, the location of the earliest atrial activation site was identified in the coronary sinus (CS) ostium in the slow-slow type and the lower atrial septum in the fast-slow type, with successful ablation achieved around it. In the present case, 1 node recorded His potentials at the 5-o’clock direction of the tricuspid valve. The tachycardia was a slow-slow-type AVNRT in the posterior AV node, with the earliest atrial excitation site at the 1-o’clock position of the mitral valve, presumed to be the left inferior extension of the conduction system.

Successful ablation was achieved at the 5–6-o’clock direction of the tricuspid valve in both the first and second sessions. In the second session, although the earliest atrial activation during tachycardia was at the 1-o’clock direction of the mitral annulus, ablation at that site was not successful, and the fragmented potential of the tricuspid valve at the 6-o’clock direction was used as an indicator. Successful ablation was achieved by energizing the tricuspid valve at the 6-o’clock fragmented potential. The presumed circuit and ablation site is shown in Figure 3G. Two previous reports of CCH and AVNRT, both in cases of situs inversus and concordant CCH, described targeting the slow pathway, with successful ablation at the posterior to midseptal mitral annulus. In our case, ablation was also successful in the vicinity of the CS ostium; it is difficult to identify the location of the slow pathway in the CCH, but it is possible to find the slow-pathway potential at the entrance of the CS as usual.

## Conclusion

We successfully performed ablation via the SVC approach for AVNRT occurring in a patient with CCH and previous modified Fontan with an EC surgery. The SVC approach to the pulmonary venous atrium after modified Fontan with an EC surgery can be a viable alternative to existing IVC methods. In the present case, slow-slow-type AVNRT in the AV node posterior to the twin AV node was successfully ablated by targeting the slow pathway and the assumed fragmented potential. This location may be helpful for slow-pathway ablation in AVNRT associated with CCH.

## Disclosures

The authors have no conflicts of interest to disclose.
