# Supplementary material for: Successful atrioventricular nodal reentrant tachycardia ablation via superior vena cava approach in a patient with crisscross heart after modified Fontan with an extracardiac conduit surgery
Source: HeartRhythm Case Rep. 2025 Jun 24;11(9):908–12. doi: 10.1016/j.hrcr.2025.06.020 (PMC12666974; doi:10.1016/j.hrcr.2025.06.020)
Supplement: Supplemental Video 2 [file mmc2.docx]

The video shows contrast injection through the conduit to delineate the anatomical relationship between the atrium and the puncture site. A transseptal puncture was performed from the superior vena cava using a BRK needle (St. Jude Medical, Inc., St. Paul, MN). Postoperative transesophageal echocardiography confirmed that the distance from the pulmonary artery to the atrium was approximately 20 mm. No pericardial effusion was observed.
